# Physicochemical, Structural, Thermal, Rheological and Functional Properties of Precooked Composite Flour from Cowpea, Unripe Plantain, and Sweet Potato, and Their Use in Cookies and Muffins

**DOI:** 10.3390/polym18212782

**Published:** 2026-11-07

**Authors:** Carmen Elena Pérez-Cervera, Daniela Colorado-Triana, Camila Andrea Suarez-Gómez, María Alejandra Castillo-Ortega, Mateo Ávila-Díaz, Claudia Denise De Paula, Ricardo David Andrade-Pizarro

**Affiliations:** 1 Agro-Industrial Engineering Program, Pontificia Bolivariana University, Montería 230002, Colombia; daniela.colorado@upb.edu.co (D.C.-T.); camila.suarez@upb.edu.co (C.A.S.-G.); maria.castilloo@upb.edu.co (M.A.C.-O.); mateo.avila@upb.edu.co (M.Á.-D.); 2 Department of Food Engineering, University of Córdoba, Montería 230002, Colombia; cdepaula@correo.unicordoba.edu.co (C.D.D.P.); rdandrade@correo.unicordoba.edu.co (R.D.A.-P.)

**Keywords:** precooked composite flour, pregelatinized starch, bakery products, sensory acceptance, pasting properties

## Abstract

Steam precooking of starch-rich raw materials yields pregelatinized biopolymer matrices; however, how the structural state of starch in multicomponent precooked flours relates to their hydration, pasting, flow, and dough and batter viscoelastic behavior remains poorly documented. This study developed and characterized a steam-precooked composite flour (PCF) from cowpea (*Vigna unguiculata*), unripe plantain (*Musa paradisiaca* cv. Hartón), and orange-fleshed sweet potato (*Ipomoea batatas* cv. Agrosavia Aurora), formulated using linear programming to meet the nutritional targets set by the “Instituto Colombiano de Bienestar Familiar” (ICBF). The PCF contained 24.08 ± 0.13% crude protein, 375.1 kcal/100 g, and 89.23 ± 0.18% in vitro protein digestibility, exceeding the ICBF minimum requirements. Differential scanning calorimetry showed no defined gelatinization endotherm, only a weak, broad endothermic region (52–92 °C; ΔH = 2.35 ± 0.56 J/g dry flour) attributed mainly to retrograded amylopectin and a minor residual starch fraction; the pasting profile lacked a viscosity peak, with breakdown indistinguishable from zero. Flour suspensions exhibited pseudoplastic behavior (shear-thinning), with a flow behavior index of 0.542 and a consistency coefficient of 0.311 Pa·sⁿ, and a flat pasting profile with negligible breakdown (0.02 cP), consistent with a collapsed structure. Cookie dough and muffin batter behaved as weak elastic gels (G′ > G″), with cookie dough approximately two orders of magnitude stiffer than muffin batter, though both showed a similar critical strain (8–13%) at the solid-to-flow transition. Baked cookies and muffins achieved high sensory acceptance and purchase intention (84% for cookies, 80% for muffins). These results suggest that PCF is a promising local alternative, with adequate nutritional, technological, and sensorial performance, to fortified blended foods based on imported ingredients for bakery products.

## 1. Introduction

Food insecurity and childhood malnutrition remain critical public health challenges in Latin America, with Colombia reporting persistent rates of stunting and micronutrient deficiency, particularly among children under five years of age in vulnerable rural and peri-urban populations [1,2]. In response, the Instituto Colombiano de Bienestar Familiar (ICBF) has historically distributed Bienestarina^®^, a fortified blended food formulated from precooked wheat, skimmed milk, and soy protein, as part of its complementary food programs targeting infants, children, and pregnant and lactating women. While Bienestarina^®^ has served as a benchmark product in Colombia for decades, its dependence on wheat imports and soy protein concentrates represents a structural vulnerability in the national food system and limits the degree to which local agricultural production chains can be valorized [3]. Colombia produces less than 1% of the wheat it consumes and imported approximately 1.9 million tons in the 2024/25 marketing year, remaining almost entirely dependent on imports [4].

Cowpea (*Vigna unguiculata* L. Walp.) is one of the most nutritionally significant grain legumes cultivated in tropical regions, particularly in sub-Saharan Africa, the Caribbean, and northeastern South America. Its seeds content 20–25 g protein/100 g (dry weight) with a favorable, lysine-rich amino acid profile that complements the limiting amino acid patterns of cereal-based staples. Additionally, cowpea is an affordable, drought-tolerant crop with well-documented potential for food security applications in low-income settings [5,6]. Soaking and dehulling substantially reduce antinutritional factors (tannins, trypsin inhibitors, phytates) that limit its bioavailability and palatability in conventional preparations [7].

Unripe plantain (*Musa paradisiaca* L. cv. Hartón) constitutes a major source of dietary starch, resistant starch, and dietary fiber across the Caribbean coast of Colombia and other tropical regions. Its starch, characterized by A-type crystallinity and high amylose content (25–30%), exhibits characteristic pasting behavior that has been extensively described in the food hydrocolloids literature [8]. Sweet potato (*Ipomoea batatas* L.) cv. Agrosavia Aurora, an export-grade orange-fleshed cultivar, contributes not only starch and dietary fiber but also substantial amounts of β-carotene, which is highly relevant for addressing vitamin A deficiency in the pediatric population [9]. The synergistic combination of these three botanically diverse sources may provide the nutritional complementarity necessary to formulate a composite flour that meets ICBF dietary specifications.

Composite flours obtained from blending two or more non-wheat flours have gained increasing interest as cost-effective, nutritionally dense alternatives to wheat-based formulations for both conventional and food security applications [10,11]. However, replacing wheat flour weakens or eliminates the gluten network and modifies the starch–water–protein interactions that govern dough behavior [12]. This behavior is governed not only by the chemical composition of the blended ingredients but also, critically, by the physicochemical state of the starch and protein fractions, parameters that are profoundly altered by any pre-processing thermal treatment [13,14]. Steam precooking, in particular, induces extensive starch gelatinization, partial protein denaturation, and cell wall disruption, collectively transforming the starch granule structure into a pregelatinized matrix whose rheological, thermal, and pasting behavior diverges substantially from that of native starch [14,15].

Pregelatinized starches are characterized by the absence of the 60–80 °C gelatinization endotherm in differential scanning calorimetry (DSC), the disruption of crystalline order detectable by X-ray diffraction and FTIR spectroscopy [15,16], and near-zero pasting viscosity in standard viscometric tests [14]. Upon cooling, gelatinized starch chains undergo retrogradation: amylopectin short chains reassociate into B-type double helices within days, while amylose chains form more thermally stable retrograded structures and inclusion complexes with endogenous lipids (V-type crystallites) that melt only above 100 °C [15,17]. These thermodynamic entities, detectable as high-temperature DSC endotherms, represent the dominant supramolecular ordering elements in precooked flour matrices and have been linked to resistance to enzymatic hydrolysis, reduced starch digestibility, and enhanced prebiotic potential [15].

Despite the nutritional and functional interest of precooked flours, systematic studies combining thermal, spectroscopic, and rheological characterization of multi-component precooked composite flours remain limited [10,11,13,18]. Closely related blends of cowpea, orange-fleshed sweet potato, and cassava or banana has been formulated for complementary feeding, but they were mainly evaluated for proximate composition, antinutritional factors, pasting properties, and sensory acceptability [6,18]. Composite flours from cowpea, sorghum, and orange-fleshed sweet potato have been characterized thermally and rheologically, but with emphasis on bioprocessing rather than on steam precooking [19,20], and pregelatinized legume flours have been applied to instant pasta and baked snacks with a focus on product development rather than on structure–rheology relationships [21]. Furthermore, the application of mathematical optimization (linear programming) to meet institutionally defined nutritional targets in composite flour design has rarely been reported for legume-based tropical composite flours [22].

The objectives of this study were: (i) to formulate, by linear programming, a composite flour from steam-precooked cowpea, unripe plantain, and sweet potato, complemented with skimmed milk powder, that meets the ICBF protein and energy targets; (ii) to characterize the structural (FTIR), thermal (DSC), hydration (swelling power and solubility), and rheological (flow and pasting) properties of the precooked composite flour (PCF); and (iii) to evaluate the viscoelastic behavior of cookie dough and muffin batter prepared with the PCF and the texture and consumer acceptance of the baked products.

## 2. Materials and Methods

### 2.1. Raw Materials

Export-grade sweet potato (*Ipomoea batatas* L.) cv. Agrosavia Aurora (orange flesh) and unripe plantain (*Musa paradisiaca* L.) cv. Hartón were procured from a local producer in Cereté, Córdoba, Colombia. Cowpea (*Vigna unguiculata* L. Walp.) cv. Caupicor 50 L042 was obtained from the University of Córdoba, Colombia. Skimmed milk powder and the other bakery ingredients (unsalted butter, white and brown sugar, eggs, vanilla essence, salt, and baking powder) were purchased from local supermarkets in Montería, Colombia.

### 2.2. Preparation of Precooked Flours

Only raw materials free of visible defects, mold, or mechanical damage were selected. Sweet potato and plantain were washed under cold running water, immersed in a 0.001% sodium hypochlorite solution for 2 min, and rinsed. After peeling, both materials were manually cut into 1 cm-thick slices and immediately immersed in a 0.3% ascorbic acid solution for 2 min to prevent enzymatic browning. The slices were then arranged in a single layer on perforated stainless-steel trays and steam-precooked at atmospheric pressure for 4 min in a steam cooker. The precooked slices were cooled to ambient temperature (25 ± 2 °C) over approximately 60 min, spread in a single layer to promote uniform cooling (uncontrolled); partial starch retrogradation may have occurred during this period.

Cowpea seeds were soaked in distilled water (1:3 *w*/*v*) for 24 h at room temperature (25 °C) to soften the seed coat and reduce antinutritional factors. After soaking, seeds were dehulled by hand rubbing under running water. The dehulled cotyledons were steam-cooked for 20 min under the same conditions described above and cooled to ambient temperature (25 ± 2 °C).

All precooked materials were dried in an industrial tray dryer (HD-20BE, Exhibir, Colombia) at 60 °C for approximately 6 h, with slices spread as a single layer on each tray (tray loading of approximately 5.25 kg/m^2^). Samples were periodically stirred (every 30 min) on the tray surface to prevent adhesion due to starch cross-linking. Samples were dried to an approximate moisture content of 8% (dry basis), as monitored gravimetrically with a moisture analyzer (Precisa Instruments Ltd., Dietikon, Switzerland). The dried materials were milled (Wondermill, Riverdale, UT, USA) and sieved through a 100-mesh sieve (<150 μm; Endecotts, London, UK). Flours were packaged in high-density polyethylene bags and stored in hermetic polypropylene containers at 28 ± 2 °C until analysis; all analyses and baking trials were carried out within two days of flour production.

### 2.3. Formulation Optimization and Preparation of the Composite Flour

The PCF formulation was optimized using the Solver add-in of Microsoft Excel 2019 (Microsoft Corp., Redmond, WA, USA) via the Simplex LP method to minimize the total cost of the formulation while simultaneously meeting the nutritional requirements established by the ICBF for supplementary feeding programs in Colombia [23]. Four ingredients were considered as decision variables: precooked cowpea flour, precooked unripe plantain flour, precooked sweet potato flour, and skimmed milk powder. The nutritional constraints were established on a 100 g formulation basis, considering target values of 21% protein, 3% fat, 63% carbohydrates, and 1.3% dietary fiber. The nutrient contribution of each ingredient was incorporated into the linear programming model according to its composition, while ingredient cost was used to define the objective function. The sum of all ingredient proportions was constrained to 100%. The linear programming model was established under the assumptions of certainty, divisibility, additivity, linearity, and non-negativity. Accordingly, the nutritional composition and cost coefficients assigned to each ingredient were treated as known and constant during optimization; ingredient proportions were considered continuous variables; the total nutrient contribution of the formulation was calculated as the additive contribution of the individual ingredients; nutrient and cost contributions were assumed to vary linearly with ingredient proportions; and all ingredient proportions were constrained to non-negative values [22]. The optimal formulation obtained was: 74% precooked cowpea flour, 6% precooked unripe plantain flour, 2% precooked sweet potato flour, and 18% skimmed milk powder. The individual flours were weighed and blended in a ribbon mixer for 10 min to ensure homogeneity.

### 2.4. Proximate Composition

The proximate composition (moisture, ash, crude protein, crude fat, crude fiber, and total carbohydrates) of the precooked composite flour (PCF) was determined in triplicate according to AOAC official methods [24]. Moisture content was measured by oven-drying at 105 °C to constant weight (AOAC 925.10); ash by incineration in a muffle furnace at 550 °C for 6 h (AOAC 923.03); crude protein by the Kjeldahl method (N × 6.25, AOAC 960.52); crude fat by Soxhlet extraction with petroleum ether (AOAC 920.39); and crude fiber by sequential acid–alkaline digestion (AOAC 962.09). Total carbohydrates were calculated by difference. Energy was estimated using Atwater conversion factors (4, 4, and 9 kcal/g for protein, carbohydrates, and fat, respectively).

Water activity (aw) was measured at 25 °C using a HygroPalm GP23 water activity meter (Rotronic, Bassersdorf, Switzerland), previously calibrated with saturated salt standards of known water activity. pH was determined potentiometrically in a 10% (*w*/*v*) aqueous suspension (AOAC 943.02), and titratable acidity by titration with 0.1 N NaOH to pH 8.2 and expressed as g lactic acid/100 g (AOAC 942.15) [24]. Color was measured in the CIELab space (illuminant D65, 10° observer) with a colorimeter (CR-10 PLUS, Konica Minolta, Osaka, Japan), calibrated with a white standard; the flour was measured in a glass cell, and the muffin crust and crumb were measured on the product surface and on a freshly cut surface, respectively. In vitro protein digestibility (IVPD) was determined by the multienzyme pH-drop method (trypsin, chymotrypsin, and peptidase; 37 °C) and calculated using Equation (1) [25]. All determinations were performed in triplicate.

(1) *IVPD*, % = 65.66 + 18.10 *x* ∆*pH*_10_
*_min_*

where ∆pH_10 min_ refers to the change in pH from initial 8.0 to the end of 10 min.

### 2.5. Microbiological Analysis

Microbiological analyses were performed on three independent samples of PCF two days after production. Molds and yeasts were enumerated according to ICMSF-2000; total coliforms were determined according to ISO 21528-2:2017 ( International Organization for Standardization. *Microbiology of the Food Chain-Horizontal Method for the Detection and Enumeration of Enterobacteriaceae-Part 2: Colony-Count Technique; ISO 21528-2:2017*; ISO: Geneva, Switzerland, 2017); *Bacillus cereus* was enumerated according to ISO 7932:2004 (International Organization for Standardization. *Microbiology of Food and Animal Feeding Stuffs-Horizontal Method for the Enumeration of Presumptive Bacillus cereus-Colony-Count Technique at 30 Degrees C*; ISO 7932:2004; ISO: Geneva, Switzerland, 2004); coagulase-positive *Staphylococcus aureus* was determined according to ISO 6888-1:2021 (International Organization for Standardization. *Microbiology of the Food Chain-Horizontal Method for the Enumeration of Coagulase-Positive Staphylococci (Staphylococcus aureus and Other Species)-Part 1: Method Using Baird-Parker Agar Medium*; ISO 6888-1:2021; ISO: Geneva, Switzerland, 2021), with confirmation by the coagulase test. *Salmonella spp*. were investigated according to ISO 6579-1:2017 (International Organization for Standardization. *Microbiology of the Food Chain-Horizontal Method for the Detection, Enumeration and Serotyping of Salmonella-Part 1: Detection of Salmonella spp*.; ISO 6579-1:2017; ISO: Geneva, Switzerland, 2017). The detection limit applied for each method was <10 CFU/g for indicator organisms; for *Salmonella* spp., results were reported as presence/absence in 25 g of sample. Results were compared with the microbiological criteria established by the Colombian Ministry of Health and Social Protection [26].

### 2.6. Swelling Power and Solubility

Swelling power and solubility were determined according to the method of Yu et al. [27] with slight modifications. Flour samples (5 g db) were dispersed in 20 mL of distilled water, thoroughly mixed, and heated at 30, 40, 50, 60, 70, 80, or 90 °C for 30 min. After cooling, the suspensions were centrifuged at 2600× *g* for 15 min. The supernatants were transferred to pre-weighed Petri dishes and dried in a hot-air oven (UN55PLUS, Memmert, Schwabach, Germany) at 110 °C to constant weight. Swelling power (SP) and solubility (S) were calculated using Equations (2) and (3), respectively.

(2) $$\mathrm{SP}=\frac{W_{t}}{W-W_{r}}$$

(3) $$S=\frac{W_{r}}{W}\times 100\%$$

where W_t_ is the weight of the wet sediment, W_r_ is the weight of the dried supernatant, and W is the weight of the sample (db).

### 2.7. Fourier-Transform Infrared Spectroscopy (FTIR)

FTIR spectra of the precooked composite flour were acquired in attenuated total reflectance (ATR) mode using an IRTRACER-100 spectrometer (Shimadzu, Kyoto, Japan). Spectra were collected over the wavenumber range 4000–400 cm^−1^ with a resolution of 4 cm^−1^, averaging 32 scans per spectrum. Three independent spectra were recorded per sample. The starch crystallinity index (ratio of absorbance at 1047/1022 cm^−1^) and the degree of ordered structure (absorbance at 995/1022 cm^−1^) were calculated to assess starch short-range molecular order [16,17].

### 2.8. Differential Scanning Calorimetry (DSC)

Thermal transitions of the PCF were characterized using a differential scanning calorimeter (DSC 250, TA Instruments, Wood Dale, IL, USA) calibrated with indium. Flour–water mixtures (1:3, *w*/*w*) were prepared in sealed vials, homogenized, and allowed to hydrate for 2 h at room temperature. Aliquots of 11.2–12.2 mg of the mixture (2.6–2.8 mg dry flour) were transferred to Tzero aluminum pans [28]. Scans were performed from 20 to 120 °C at 10 °C/min under nitrogen purge (50 mL/min). Two independent replicates were measured per sample. Onset temperature (T_on_), peak temperature (T_p_), conclusion temperature (T_c_), and the total transition enthalpy (ΔH, J/g dry flour) were determined from the resulting thermograms.

### 2.9. Rheological Characterization

#### 2.9.1. Flour Dispersions—Flow Curve

The flow behavior of PCF aqueous dispersions was characterized using a rotational rheometer (Anton Paar MCR 302, Graz, Austria) equipped with a parallel-plate geometry (diameter: 30 mm; gap: 1 mm). Dispersions at 8 g/100 mL were hydrated for 30 min at room temperature before measurement, and the temperature was kept at 25.0 ± 0.1 °C with a Peltier system. Flow curves were obtained in controlled-shear-rate mode by applying a linear ascending ramp from 0.1 to 100 s^−1^ followed by a linear descending ramp from 100 to 0.1 s^−1^. Fifty points were recorded per ramp. The power law model (σ = K × γ̇^ⁿ^) was fitted to the data using non-linear regression, where σ is shear stress (Pa), γ̇ is shear rate (s^−1^), K is the consistency coefficient (Pa × s^ⁿ^), and n is the flow behavior index (dimensionless). Goodness of fit was evaluated by the coefficient of determination (R^2^) and the root mean square error (RMSE). Time-dependent (thixotropic) behavior was assessed by comparing the K and n values of the ascending and descending curves with paired *t*-tests. Three independent replicates were performed per sample.

#### 2.9.2. Pasting Properties

Pasting profiles of the PCF were determined in triplicate using a rotational rheometer (Anton Paar MCR 302, Graz, Austria) equipped with a vane spindle (Anton Paar ST24-2D/2V/2V-30, Graz, Austria). Flour suspensions (8 g/100 mL, dry weight basis) were prepared in distilled water and subjected to the following temperature program: equilibration at 50 °C for 1 min, heating from 50 to 95 °C at 12 °C/min, holding at 95 °C for 5 min, cooling from 95 to 50 °C at 12 °C/min, and holding at 50 °C for 2 min, with constant stirring at 160 rpm. Peak viscosity, trough viscosity, breakdown, final viscosity, setback, and pasting temperature were determined from each profile.

#### 2.9.3. Amplitude Sweeps of Cookie Dough and Muffin Batter

Cookie dough and muffin batter doughs prepared with PCF were characterized by small-amplitude oscillatory shear using the same parallel-plate geometry (30 mm diameter; gap: 2 mm). The exposed sample surface was covered with low-viscosity silicone oil to prevent moisture loss. Strain amplitude was varied from approximately 0.007 to 50% at an angular frequency of 6.28 rad/s (1 Hz) and 25 °C (Peltier control). The limit of the linear viscoelastic region (γ_L_) was defined as the strain at which G′ deviated by more than 5% from its plateau value, and the flow point (γ_f_) as the strain at which G′ = G″ [29]; these two parameters were evaluated separately. Measurements were performed on three independent preparations of each system.

### 2.10. Preparation of Bakery Products

#### 2.10.1. Preparation of Cookies

The cookies were prepared following the experimental process described by Mouafo et al. [30], with some modifications. The unsalted butter (19.6%), brown sugar (12%) and white sugar (9%) were beaten until a homogeneous, smooth and lump-free mixture was obtained. Egg (14%) and vanilla essence (0.75%) were added to the creamed mixture, and blended. Next, the PCF (44%), salt (0.15%) and baking powder (0.5%) were premixed and then incorporated into the creamed mixture. After homogenization, the dough was rolled out on a stainless-steel table with a wooden rolling pin to a thickness of 1.4 cm, cut with a circular cutter (diameter: 3.5 cm) and baked at 180 °C for 18 min in an oven (Tornado, Pallomaro, Cali, Colombia). The cookies were cooled to room temperature (25 °C) and stored in hermetically sealed glass jars prior to analysis. Cookies were produced in three independent batches, and texture and sensory analyses were performed within 24 h of baking.

#### 2.10.2. Preparation of Muffins

The preparation of the muffins was carried out following the methodology described by Ha et al. [31], with some modifications in the beating conditions and baking time, defined through preliminary tests. Initially, the unsalted butter (18%) at room temperature and the white sugar (11%) were beaten until a creamy, homogeneous mixture was obtained. Egg (27%) and vanilla essence (1%) were added to the creamed mixture, and stirring was continued for 3 min. Then, the PCF (31%), salt (0.3%) and baking powder (0.3%) were premixed and added to the creamed mixture along with water (11.4%). The mixture was beaten for 4 min, until a soft, homogeneous, and aerated batter was obtained. The batter was distributed in portions of 12 g in size No. 2 baking cups (base diameter: 4 cm; height: 2.5 cm), placed in stainless-steel muffin molds. The muffins were baked in an oven (Tornado, Pallomaro, Colombia) at 170 °C for 15 min. The muffins were cooled to room temperature (25 °C) and stored in hermetically sealed glass jars prior to analysis. Muffins were produced in three independent batches, and texture and sensory analyses were performed within 24 h of baking.

### 2.11. Texture and Sensory Analysis of Cookies and Muffins Made with Precooked Composite Flour

#### 2.11.1. Texture Analysis

Instrumental texture was evaluated using a Brookfield CT3 Texture Analyzer (Brookfield Engineering, Middleboro, MA, USA) equipped with a cylindrical probe (diameter: 30 mm). Muffin samples were compressed twice at 30% deformation (trigger force: 5 g; test speed: 1 mm/s; post-test speed: 10 mm/s). The texture profile analysis (TPA) parameters calculated were hardness (N), cohesiveness, and springiness [32]. The breaking force of the cookies was determined using a three-point bending test. Each cookie was placed on two supports spaced 30 mm apart and loaded at its center by an upper blade at a speed of 60 mm/min until fracture occurred. The maximum force at the moment of fracture was recorded as the breaking force (N). At least six replicates per product type were analyzed.

#### 2.11.2. Sensory Evaluation

Consumer acceptance of PCF products was evaluated by a hedonic sensory test at the University of Córdoba (Montería, Colombia), approved by the institutional ethics committee of the University of Córdoba in its session of 18 March 2024. All participants gave written informed consent before the test. One hundred untrained panelists (age range: 25–35 years; 56% female) were recruited with written informed consent. Inclusion criteria were being a regular consumer of cookies and muffins; exclusion criteria were self-reported allergy or intolerance to milk, egg, legumes, or any other ingredient of the products, which were disclosed to participants beforehand. Each participant evaluated one cookie (10 g) and one muffin (10 g), coded with random three-digit numbers and served monadically at room temperature in a randomized, balanced order, with water for palate cleansing between samples. Panelists scored appearance, overall impression, texture, and flavor using a 9-point hedonic scale; and purchase intent was assessed with a 5-point scale. All sessions were conducted in individual booths under white fluorescent lighting at 25 °C, between 09:00 and 11:00 h.

### 2.12. Statistical Analysis

All data are expressed as mean ± standard deviation from at least three independent replicates. The experimental unit was the independently prepared sample: the PCF was analyzed in triplicate, and baked products were evaluated on three independent batches. The effect of temperature on swelling power and solubility was evaluated by one-way analysis of variance (ANOVA) followed by Tukey’s honestly significant difference test (*p* < 0.05), after verifying the normality of residuals (Shapiro–Wilk test) and the homogeneity of variances (Levene’s test). Power Law parameters were estimated by non-linear least squares as described in Section 2.9.1. Statistical analyses were performed using JMP^®^ Pro 17 (SAS Institute Inc., Cary, NC, USA).

## 3. Results and Discussion

### 3.1. Physicochemical, Structural, Thermal and Functional Characterization of Precooked Flour

#### 3.1.1. Proximate Composition of Precooked Composite Flour

Moisture content of the PCF was 7.93 ± 0.03%, within the limits recommended for shelf-stable composite flours (<10%). The PCF showed a low water activity (a_w_ = 0.477 ± 0.005), below 0.60, the threshold below which the growth of bacteria, yeasts, and molds is generally inhibited [10], indicating a favorable condition for microbiological stability during ambient storage. Crude protein content (24.08 ± 0.13%) exceeded the protein content specified by ICBF for Bienestarina Más (21 g/100 g) [23], reflecting the dominant contribution of the 74% precooked cowpea fraction [5]. Crude fat (5.59 ± 0.19%), ash (3.76 ± 0.05%), and crude fiber (1.53 ± 0.26%) were within ranges reported for legume–cereal composite flours [10,11]. Available carbohydrates were 57.11%, yielding an estimated energy value of 375.1 kcal/100 g (Atwater factors), above the 360 kcal/100 g specified for Bienestarina Más [23]. In vitro protein digestibility was 89.23 ± 0.18%, higher than values reported for raw cowpea flour (60–75%) [5] and comparable to that of casein (87–90%), This result is consistent with the reduction in heat-labile antinutritional factors, such as trypsin inhibitors, by soaking, dehulling, and steam precooking [5,6]. pH (6.44 ± 0.04) was near-neutral and titratable acidity (0.182 ± 0.010%) was low, similar to values reported for legume based complementary food blends [6]. Flour color (L* = 89.97 ± 0.06; a* = 3.02 ± 0.03; b* = 19.13 ± 0.06) indicated a pale cream-yellow powder; the yellowness may be due to the combined contribution of sweet potato carotenoids [9], cowpea cotyledon pigments, the milk powder, and mild non-enzymatic browning during drying.

#### 3.1.2. FTIR Spectroscopy of Precooked Composite Flour

**Figure 1:**
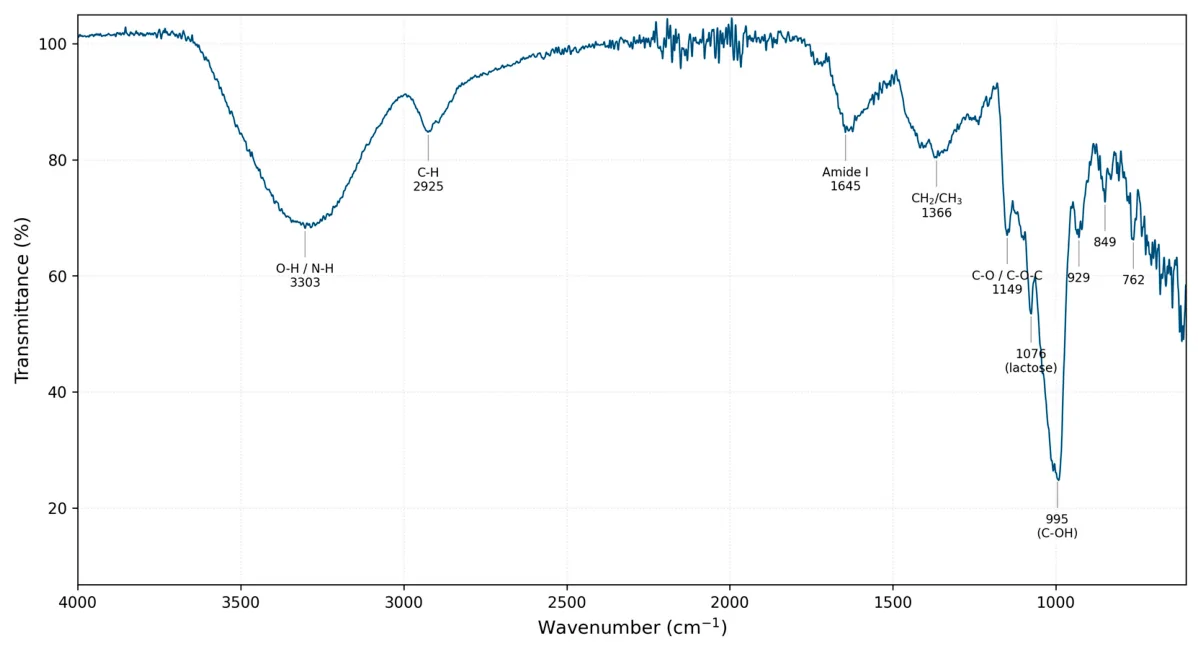
Fourier transform infrared spectroscopy (FTIR) spectra of precooked composite flour.

Figure 1 shows the FTIR spectrum of the precooked composite flour, in which the coexistence of the functional groups characteristic of its components was evident: starch provided by the precooked flours of cowpea, unripe plantain and sweet potato; vegetable and dairy proteins; and the lipids and lactose of the milk powder.

The broad band centered at 3303 cm^−1^ corresponds to O-H stretching coupled with N-H vibrations (amide band A) and reflects the contribution of hydroxyl groups from cowpea, unripe plantain, and sweet potato starch, and amino groups from cowpea and milk powder proteins [20,33]. The broadening of this band is characteristic of matrices with a high capacity for hydrogen bonding, typical of flours rich in carbohydrates and proteins [34]. The band at 2925 cm^−1^ corresponds to the asymmetric C-H stretching of the methylene and methyl groups, mainly due to the lipid fractions of the milk powder and cowpea [35,36].

The band observed at 1645 cm^−1^ is assigned to the amide I region (C = O stretching of the peptide bond) due to the presence of both plant (cowpea) and dairy proteins (casein and β-lactoglobulin) [37,38]. Its position lies in the region usually associated with unordered and β-sheet structures, which would be compatible with proteins denatured by precooking [20,36]; however, in a multicomponent flour the amide I envelope overlaps with the O–H bending of water (~1640 cm^−1^) and includes contributions from several proteins [38]. The band at 1366 cm^−1^ is associated with the symmetrical deformation of the CH_2_/CH_3_ groups and in-plane O-H bending, while the bands at 929, 849, and 762 cm^−1^ correspond to vibrations of the glucopyranose ring, consistent with the preservation of the glucopyranose ring after thermal processing [34,39].

The 900–1200 cm^−1^ region exhibits the highest spectral intensity, because starch is the major component of the mixture. The most intense band was located at 995 cm^−1^ and is attributed to the C-OH bending of the C6 carbon of the glucose unit, a band recognized as a marker sensitive to the short-range structural order of starch [40,41]. The adjacent bands at 1076 and 1149 cm^−1^ correspond to C-O stretching of the anhydroglucose backbone and C-O-C glycosidic bonds, with an additional contribution from milk powder lactose, whose characteristic peak also appears near 1076 cm^−1^ [35,36].

The PCF exhibited an R1047/1022 ratio of 0.62 ± 0.05 and an R995/1022 ratio of 1.30 ± 0.4. The low R1047/1022 value suggests weak short-range order, which may result from the loss of native granular organization during steam precooking, since gelatinization reduces this ratio, while retrogradation increases it [16,42]. The high R995/1022 value may reflect partial reassociation of starch chains during cooling and drying, consistent with the weak retrogradation endotherm detected by DSC. However, lactose from the milk powder and cowpea cell wall polysaccharides also absorb in the 1000–1080 cm^−1^ region.

#### 3.1.3. Thermal Properties of Precooked Composite Flour

The thermograms of the PCF showed no defined gelatinization endotherm. Only a very weak and broad endothermic region was observed between 52.5 ± 0.4 and 92.1 ± 0.4 °C, comprising two shallow, overlapping peaks at 63.5 ± 0.6 °C and 82.4 ± 1.0 °C, with a total enthalpy of 2.35 ± 0.56 J/g dry flour. In contrast, raw flours from the same botanical sources exhibit well-defined gelatinization endotherms, with peak temperature (Tp) values of 76–82 °C for cowpea [43,44], 70–80 °C for sweet potato [45], and 72–84 °C for unripe plantain [46,47,48]; native cowpea flour also displays a protein denaturation endotherm at ≈88 °C [44].

The first peak (≈63.5 °C) falls within the temperature range in which amylopectin crystallites formed by retrogradation melt (≈40–65 °C) [15,49], and the second peak (≈82.4 °C) coincides with the gelatinization range of native cowpea starch and may correspond to a minor fraction of starch that remained ungelatinized after steam precooking, probably inside intact cotyledon cells, where the cell wall restricts water access and swelling [50], with a possible contribution from residual native proteins (cowpea globulins or whey proteins from the milk powder) [44,51]. The low enthalpy and the absence of a defined gelatinization endotherm, together with the pasting profile without a viscosity peak (Section 3.2.2), may indicate that steam precooking gelatinized most of the starch in the PCF, whereas cooling and drying promoted a limited reassociation of amylopectin.

#### 3.1.4. Functional Properties of the Precooked Composite Flour

**Figure 2:**
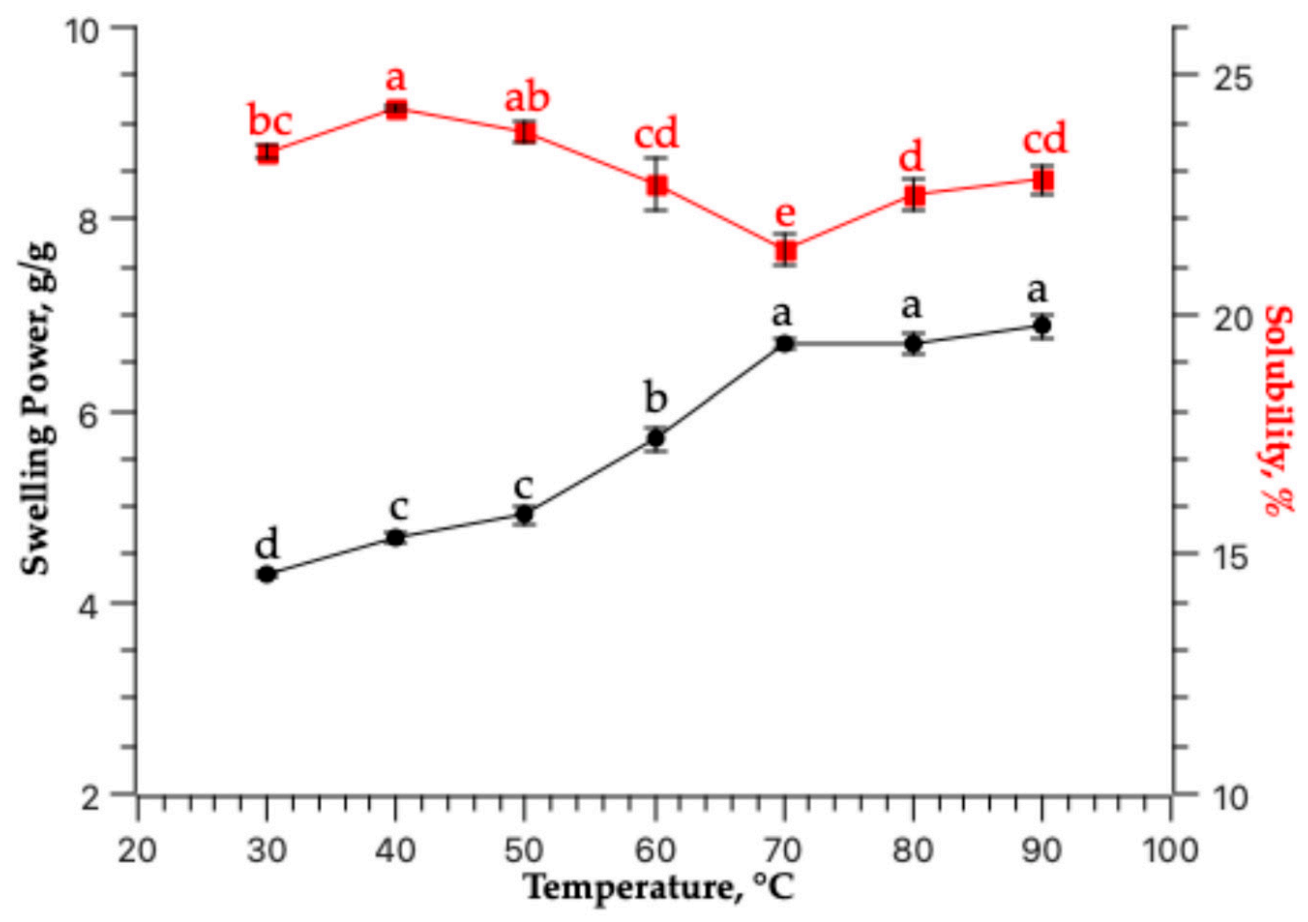
Swelling power and solubility of precooked composite flour.

The swelling power (SP) and solubility of the PCF at temperatures ranging from 30 to 90 °C are shown in Figure 2. SP values of the composite flour were in the range of 4.29–6.88 g/g, which are lower than those reported for isolated starches from sweet potato (7–12 g/g) and unripe plantain (8–14 g/g) [14,52,53]. This difference may be attributed to the restrictive effect of cowpea proteins, which form complexes with starch chains through hydrophobic interactions and hydrogen bonding, thereby reducing granular expansion [54], and to the presence of insoluble dietary fiber contributed by precooked unripe plantain, which competes with starch for the free water available in the system [55]. In addition, the starch of the PCF had already been gelatinized and partially reassociated during precooking and drying, which limits its re-swelling capacity compared with native starch granules [56,57]. Steam precooking can cause collapses in the granular architecture and limits subsequent re-swelling, an important functional characteristic of pregelatinized flours.

The analysis of variance shows that temperature significantly affected the swelling power (*p* < 0.0001). SP of the composite flour increased from 4.29 g/g at 30 °C to 6.69 g/g at 70 °C, and then remained practically constant up to 90 °C (6.69–6.88 g/g). The most pronounced increase, occurring within the 60–70 °C interval, may be due to several factors: (i) the melting of weak retrograded amylopectin crystallites formed after precooking, which typically melt between about 40 and 70 °C and release chains able to bind water [15,58]; (ii) the gelatinization of a minor starch fraction that may have remained ungelatinized after the short steam-precooking step, particularly inside intact cowpea cotyledon cells [50]; and (iii) the thermal unfolding of whey proteins from the milk powder (70–80 °C) [59]. Composite flour possesses a functional and stable water retention capacity at processing temperatures above 70 °C, a desirable characteristic for its application in baked goods, extruded products, and instant soups.

ANOVA shows that solubility was significantly affected by temperature (*p* = 0.0043). Solubility was high, ranging from 21.35 ± 0.34% (70 °C) to 24.31 ± 0.05% (40 °C). A large part of this soluble fraction can be attributed to non-starch components: the skimmed milk powder (18% of the blend) contains approximately 50% lactose plus soluble whey proteins and minerals, and cowpea contributes soluble sugars and α-galactosides. These values are higher than those reported for refined cereal flours (8–15%) and native starches (<5%) under similar conditions [14,60,61], which may favor the dispersibility of the flour in aqueous systems and its application as a thickening agent or in functional beverage formulations.

Solubility of the precooked composite flour exhibited a biphasic behavior as a function of temperature, decreasing from a maximum of 24.31% at 40 °C to a minimum of 21.35% at 70 °C, followed by a slight recovery to approximately 22.67% between 80 and 90 °C. This profile was inverse to that of swelling power. The high solubility at low temperatures (30–50 °C) may be attributed to the preferential leaching of low molecular weight amylose fractions, soluble proteins, and free sugars before gelatinization [61,62]. The subsequent decrease between 40 and 70 °C may result from macromolecular reorganization during heating, in which amylose and amylopectin chains become entrapped within the swollen gel matrix, while the protein fraction acts as a physical barrier limiting diffusion [63,64]. Similar behavior has been reported in composite flours from legumes and tubers, in which the protein fraction acts as a physical barrier that delays the diffusion of soluble polysaccharides out of the granule [65,66].

The slight rebound above 80 °C may reflect the partial rupture of overcooked granules and the subsequent release of high-molecular-weight amylopectin fragments into the aqueous medium, a phenomenon also attributable to the onset of thermal dissociation of protein–starch complexes [62,67]. This combination of moderate SP and high solubility suggests that the flour may be suitable for porridge and beverage applications requiring rapid hydration and smooth texture.

#### 3.1.5. Microbiological Safety of the Precooked Composite Flour

The PCF showed molds and yeasts at 100 CFU/g, total coliforms below 10 CFU/g, presumptive *Bacillus cereus* and *Staphylococcus aureus* (coagulase-positive) below the detection limits, and absence of *Salmonella* spp. in 25 g in all three samples. These results comply with the Colombian microbiological criteria for food category [26]; however, the mold and yeast count was at the upper acceptance limit (100 CFU/g), indicating that hygienic control of the post-drying operations (milling, sieving, and packaging) should be improved. Because the analyses were performed shortly after production, these results describe the initial microbiological quality of the PCF and do not establish its safety throughout storage, which must be confirmed in shelf-life studies.

### 3.2. Rheological Properties of the Precooked Composite Flour

#### 3.2.1. Flow Curves of Flour Dispersions

**Figure 3:**
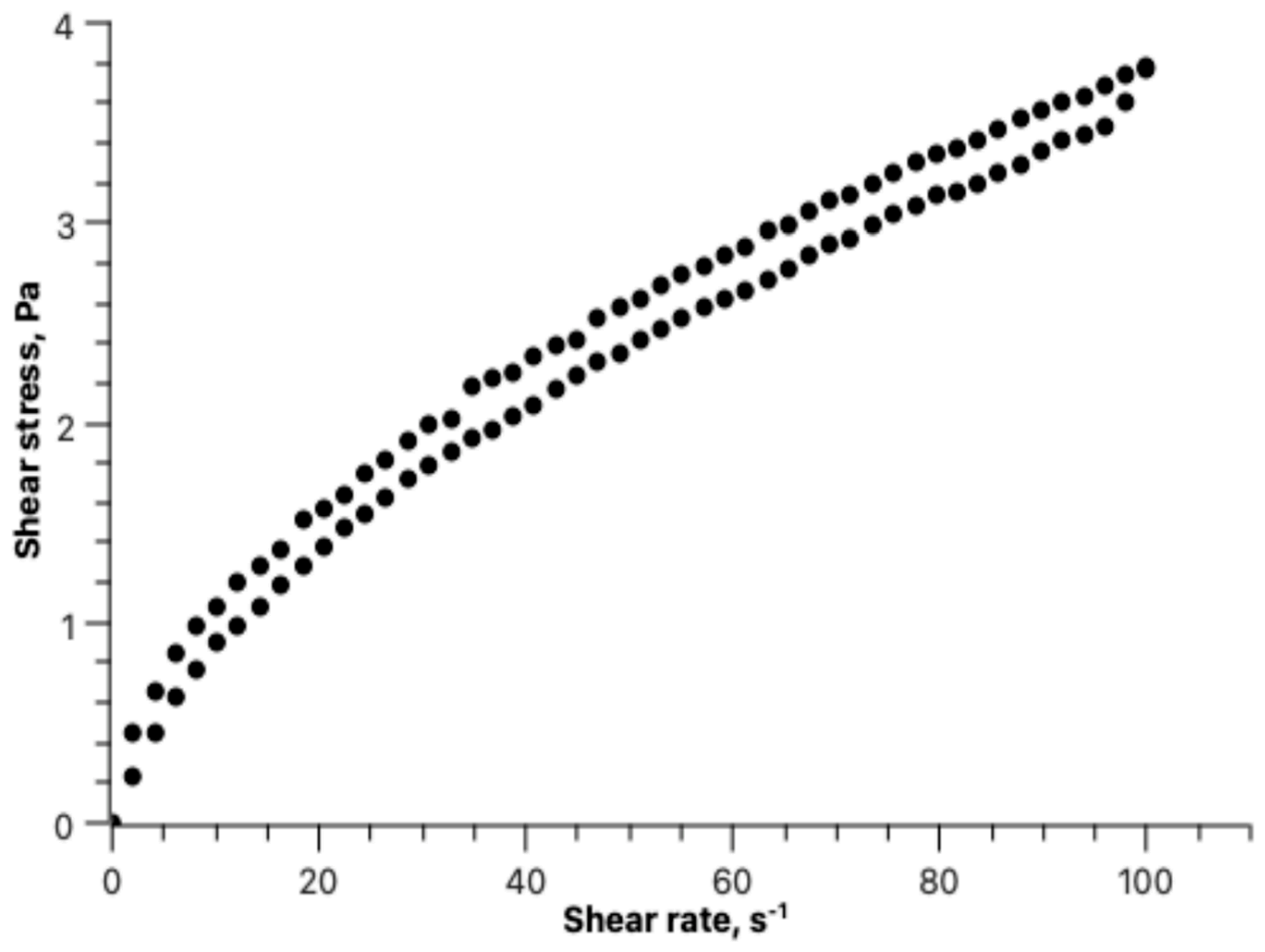
Rheograms of precooked composite flour.

Figure 3 shows that the ascending and descending curves coincide; there is no hysteresis phenomenon, which indicates that the suspensions exhibited time-independent behavior. Student’s *t*-test for the rheological data showed no significant differences (*p* = 0.377) between the values obtained from the upward and downward curves, confirming that the rheological behavior of the suspension is time-independent. PCF dispersions exhibited non-Newtonian, shear-thinning (pseudoplastic) behavior over the applied shear rate range of 0.1–100 s^−1^; this behavior has been reported for flours from cowpea, sorghum and orange fleshed sweet potato [19]. The rheological data were fitted to a Power Law model (R^2^ > 0.99; RMSE = 0.01), with a consistency coefficient of 0.311 ± 0.021 Pa·sⁿ and a flow behavior index of 0.542 ± 0.013, confirming the pseudoplastic behavior of this flour. These values are similar to those reported for sweet potato flour dispersions [68]. PCF dispersions are structured with pseudoplastic behavior, which is advantageous for food applications as it allows easy dispensing at high shear rates while maintaining structural cohesion at rest.

#### 3.2.2. Pasting Properties of the Precooked Composite Flours

**Figure 4:**
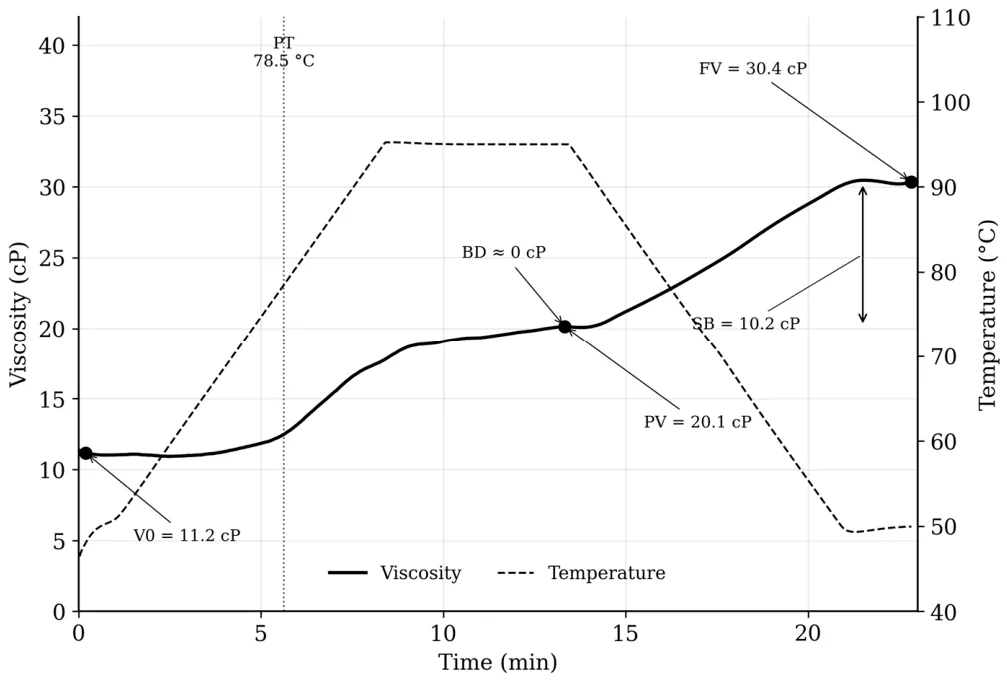
Pasting properties of precooked composite flour.

The pasting profile obtained for the precooked composite flours is shown in Figure 4. The composite flour exhibited a profile without a defined peak during heating or retention at 95 °C, with a gradual increase in viscosity that is most pronounced during cooling.

Table 1 shows the pasting parameters for the precooked composite flour. The very low initial viscosity of the PCF (11.2 cP) and its minimal response during heating are consistent with extensive pregelatinization of the starch during the steam precooking of the three plant materials. In raw matrices, native starch progressively absorbs water upon heating, and upon exceeding the gelatinization temperature, the granules swell, generating a characteristic viscosity peak [69,70]. In the PCF, this peak is absent because the granules have lost their swelling power after the disruption of their crystalline structure during steam precooking [71,72]. This behavior has been described for pregelatinized rice, corn, and sweet potato, in which the viscosity decreases or remains flat during heating and increases only during cooling [73,74].

The apparent pasting temperature (78.5 °C) of the composite flour does not represent classic gelatinization but rather the point at which the solubilization of pregelatinized amylose and the progressive hydration of proteins begin to generate a detectable change in viscosity. Its shift toward higher temperatures in pregelatinized flours may be attributed to interactions between amylose, proteins, and endogenous lipids that restrict the availability of free water [72].

The precooked composite flour showed a breakdown close to zero (0.02 cP); the difference between peak and trough viscosities was far below the standard deviations of both parameters. This result is consistent with the absence of a viscosity peak and indicates that the PCF did not contain an appreciable fraction of intact swollen granules that could rupture under heat and shear in the conditions tested (8 g/100 mL, 95 °C, 160 rpm). Given that the overall viscosities were very low (11–30 cP), the absence of breakdown indicates the stability of a low-viscosity system [75]. The moderate increase in viscosity during cooling (setback = 10.2 cP) indicates a limited tendency to form firm gels. These features, together with the high cold-water solubility, may be useful in reconstitutable beverages, instant snacks, and foods that require rapid preparation without additional cooking [21,72].

#### 3.2.3. Dynamic Oscillatory Rheology of Cookie Dough and Muffin Batter

**Figure 5:**
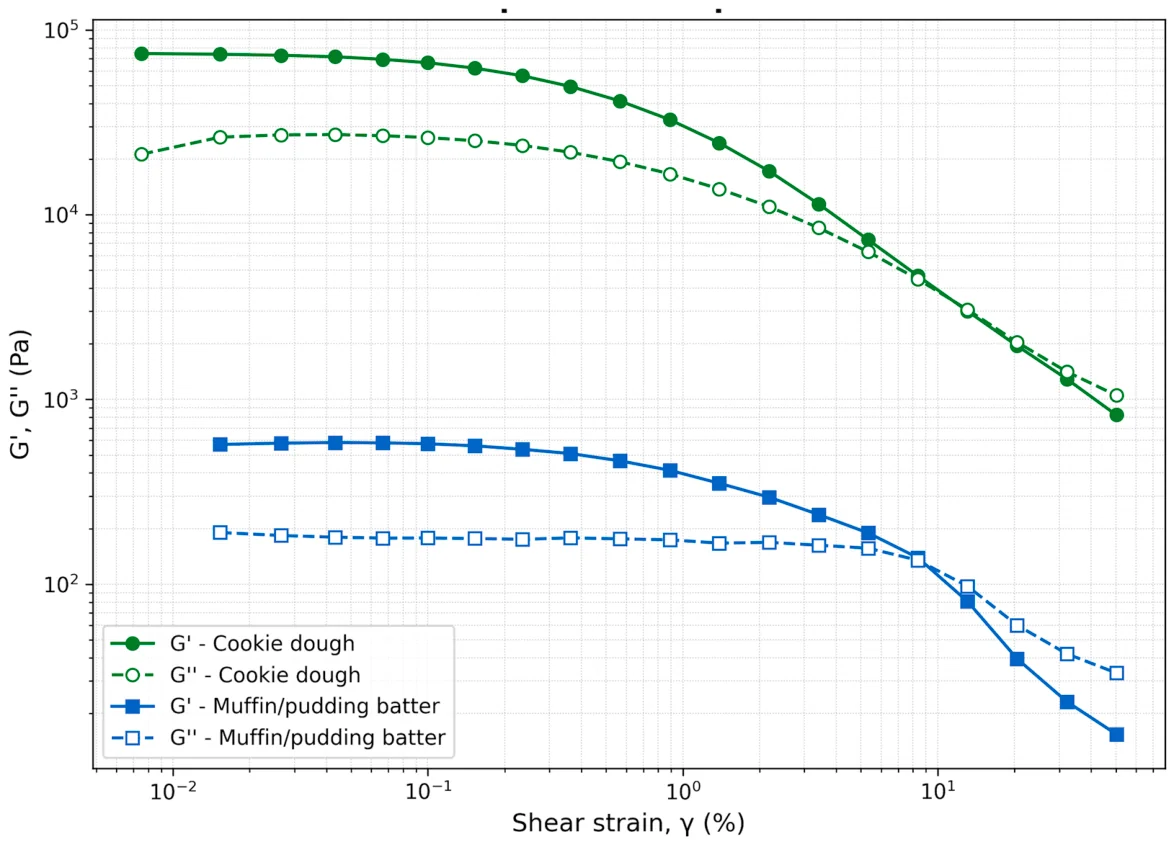
Amplitude sweep test results for cookie dough and muffin batter prepared from precooked composite flour.

Small-amplitude oscillatory shear tests showed that the cookie dough and muffin batter prepared from the precooked composite flour behaved as weak, predominantly elastic gels within their linear viscoelastic region, with storage modulus (G′) exceeding loss modulus (G″) throughout the plateau (Figure 5), a signature of the starch–protein network typically formed by fermented or precooked legume–tuber flour blends [19]. However, the cookie dough showed a G′ plateau (71,000–75,000 Pa) almost two orders of magnitude above the muffin batter plateau (570–580 Pa). The large difference in moduli is mainly attributed to formulation: the cookie dough contains much less water and more PCF than the muffin batter, and the two systems also differ in fat and sugar contents. Similar differences have been reported for muffin batters containing brewers of spent grain and milk thistle seed proteins [76,77]. Despite this difference in absolute moduli, both systems showed a comparable critical strain (8–13%), at which G″ overtook G′, marking the onset of network breakdown and the transition from solid-like to flow behavior; this convergence suggests that the starch–protein–fiber architecture imparted by the cowpea, sweet potato and unripe plantain fractions is fragile, although water content and mixing conditions dictate their absolute consistency [78].

### 3.3. Baked Products Made from Precooked Composite Flour

#### 3.3.1. Cookies

The cookies fractured at a breaking force of 0.126 ± 0.018 N, a value considerably lower than those reported for corn-based, gluten-free cookies evaluated via three-point bending tests (18–51 N) [79], indicating a very fragile, short texture that breaks with little force. This behavior may be due to the absence of a continuous gluten network, the high fat content of the formulation (19.6% butter), which limits the formation of a cohesive matrix, and the low structuring capacity of the pregelatinized starch after baking.

Consumer acceptance scores for PCF cookies showed high values (6.5/9.0), with an appearance score of 7.35 ± 1.23, an overall impression score of 7.33 ± 1.16, a taste score of 7.17 ± 1.63, and a texture score of 6.85 ± 1.75, placing them in the “I like moderately” to “I like very much” range. These scores exceeded the commonly applied acceptability threshold of 6.0. Eighty-four percent of panelists indicated their willingness to purchase the PCF cookie, a higher percentage than that obtained for legume-based gluten-free cookies, which reported between 65% and 80% [80]. The high purchase intention rate may be due to an acceptable taste profile and a visually appealing appearance score.

#### 3.3.2. Muffins

PCF muffins showed a hardness of 29.52 ± 4.69 N, within the range reported for legume-fortified muffin formulations [32]. Cohesiveness (0.300 ± 0.09) and springiness (0.367 ± 0.058) were low, indicating a crumbly crumb with limited elastic recovery, as frequently observed in gluten-free muffins [76]. PCF enabled the production of two products with distinct textures, suggesting its versatility as an ingredient in different bakery formulations.

The lightness of the crust (L* = 57.7 ± 6.9) and the crumb (L* = 38.30 ± 0.656) of muffins made from the composite flour were different, with the crust being considerably lighter and more variable, likely reflecting localized surface dehydration and non-uniform heat exposure during baking, a pattern reported for muffins made from legume-supplemented composite flours [78]. Both parts exhibited positive a* (10.6 ± 1.6 crust; 5.57 ± 0.231 crumb) and high b* values (37.05 ± 5.0 crust; 22.17 ± 0.929 crumb), indicating a dominant red-yellow hue. The higher a* and b* values of the crust are mainly attributed to Maillard reactions and sugar caramelization at the surface during baking [81]. The yellowness of the crumb may be attributed to egg yolk pigments and sweet potato carotenoids, which increase a* and b* values in composite flours and breads [82,83].

Sensory evaluation of the muffins prepared from precooked composite flour showed generally favorable acceptance across all attributes, with flavor receiving the highest score (7.6 ± 1.27), followed by global appearance (7.46 ± 0.97) and global impression (7.39 ± 1.02), values consistent with the “I like moderately” to “I like very much” range; these ranges coincide with those obtained for other sweet potato–cowpea-based composite blends [18]. Texture was the lowest-rated attribute (6.57 ± 1.59); legumes rich in fiber and protein (cowpea) and the fractions of unripe fruit (unripe plantain) alter crumb cohesiveness and mouthfeel more markedly than they affect flavor or appearance, making texture the most sensitive quality parameter in composite-flour baked products [78]. Despite this lower textural score, strong liking for flavor and appearance translated into robust purchase intention, with 80% positive intention (45% would definitely buy the product and 35% probably would).

## 4. Conclusions

Steam precooking, combined with a linear-programming-based formulation, produced a composite flour from cowpea, unripe plantain, sweet potato, and skimmed milk powder that met the ICBF protein and energy targets and showed high in vitro protein digestibility. This flour is a technologically sound, locally sourced alternative to wheat- and soy-based Bienestarina^®^. The absence of a pasting peak, a breakdown indistinguishable from zero, and the very low enthalpy measured by DSC in excess water (2.35 ± 0.56 J/g dry flour, without a defined gelatinization endotherm) suggest partial reassociation of starch into thermally stable structures, which may be attributed to retrograded amylose and/or amylose–lipid complexes. The flour formed shear-thinning dispersions and retained enough structuring capacity to produce a stiff cookie dough and a soft muffin batter, both predominantly elastic at small strains, with a linear viscoelastic limit below 0.3% strain and a flow point at 8–13% strain. The composite flour can generate two functionally distinct doughs: an elastic, high-modulus cookie dough and a softer, aerated muffin batter. Cookies and muffins produced from the PCF achieved sensory acceptance scores above the standard acceptability threshold, translating into high consumer purchase intention. Taken together, these findings support the technical and sensory viability of PCF as a multifunctional ingredient for bakery, and instant-food applications. Future work should address pilot-scale processing and shelf-life stability under real storage conditions.

## Figures and Tables

**Table 1:** Pasting parameters of the composite precooked flours.

Parameters	Composite Precooked Flours
Initial Viscosity (cP)	11.2 ± 0.8
Pasting Temperature (°C)	78.5 ± 0.6
Peak Viscosity (cP)	20.1 ± 1.1
Trough Viscosity (cP)	20.1 ± 0.8
Breakdown (cP)	0.02 ± 0.01
Final Viscosity (cP)	30.4 ± 1.7
Setback (cP)	10.2 ± 1.2

## Data Availability

The original contributions presented in this study are included in the article. Further inquiries can be directed to the corresponding author.
